# High ankle brachial index predicts high risk of cardiovascular events amongst people with peripheral artery disease

**DOI:** 10.1371/journal.pone.0242228

**Published:** 2020-11-12

**Authors:** Jonathan Golledge, Joseph V. Moxon, Sophie Rowbotham, Jenna Pinchbeck, Frank Quigley, Jason Jenkins

**Affiliations:** 1 Queensland Research Centre for Peripheral Vascular Disease, College of Medicine and Dentistry, James Cook University, Townsville, Queensland, Australia; 2 Centre for Molecular Therapeutics, The Australian Institute of Tropical Health and Medicine, James Cook University, Townsville, Queensland, Australia; 3 The Department of Vascular and Endovascular Surgery, Townsville University Hospital, Townsville, Queensland, Australia; 4 School of Medicine, University of Queensland, Brisbane, Queensland, Australia; 5 Mater Hospital, Townsville, Australia; 6 Department of Vascular and Endovascular Surgery, Royal Brisbane and Women’s Hospital, Herston, Queensland, Australia; NIHR Leicester Biomedical Research Centre, UNITED KINGDOM

## Abstract

Ankle-brachial pressure index (ABPI) is commonly measured in people referred to vascular specialists. This study aimed to assess the association of high ABPI (≥ 1.4) with cardiovascular events in people with peripheral artery disease (PAD). 1533 participants with PAD diagnosed by a vascular specialist were prospectively recruited from four out-patient clinics in Australia. ABPI was measured at recruitment and the occurrence of myocardial infarction (MI), stroke or cardiovascular death (major cardiovascular events; MACE) and any amputation were recorded over a median (inter-quartile range) follow-up of 3.3 (1.0–7.1) years. The association of high, compared to normal, low (0.5–0.9) or very low (<0.5), ABPI with clinical events was estimated using Cox proportional hazard analyses, adjusting for traditional risk factors and reported as hazard ratio with 95% confidence intervals. 596 (38.9%), 676 (44.1%), 157 (10.2%) and 104 (6.8%) participants had normal, low, very low and high ABPI, respectively. Participants with high ABPI had increased risk of MACE, MI and death by comparison to those with either normal ABPI [1.69 (1.07, 2.65), 1.93 (1.07, 3.46) and 1.67 (1.09, 2.56)] or either low or very low ABPI [1.51 (1.02, 2.23), 1.92 (1.16, 3.19) and 1.47 (1.02, 2.14)] after adjusting for other risk factors. Findings were similar in a sensitivity analysis excluding people with ABPI only measured in one leg (n = 120). Participants with high ABPI also had an increased risk of MACE and MI compared to those with very low ABPI alone. High ABPI is a strong indicator of excess risk of cardiovascular events amongst people with PAD.

## Introduction

Ankle-brachial pressure index (ABPI), the ratio between systolic pressure at the ankle and arm, is recommended by current guidelines as a diagnostic test for peripheral artery disease (PAD) [1, 2]. Low ABPI (usually defined as ≤0.9) is a strong predictor of increased risk of myocardial infarction (MI), stroke and cardiovascular death and has been reported to improve cardiovascular event prediction over conventional models, such as the Framingham risk score, alone [3–6].

Population studies report that about 3 to 5% of people have an abnormally high ABPI, commonly defined as ≥1.4 [3, 7–9]. In these instances, the test is typically concluded to not be able to provide diagnostic information about PAD and it is currently unclear how such patients should be managed [1, 2, 10]. Since high ABPI indicates arterial calcification, an established predictor of cardiovascular events, it is likely that its identification has important implications for prognosis and treatment [11]. In previous studies high ABPI has been associated with increased risk of cardiovascular events in some but not all studies [3, 7–9, 12–14]. Mostly these studies have investigated community populations that have a low prevalence of cardiovascular disease [7–9, 12, 13]. A recent meta-analysis suggested that high ABPI may also be an important prognostic indicator for cardiovascular events in people with established cardiovascular disease [13].

PAD includes a group of occlusive and aneurysmal diseases, such as lower limb artery occlusion, abdominal aortic aneurysm (AAA) and carotid artery disease, resulting from narrowing and occlusion of the peripheral arteries, which is associated with a particularly high rate of major cardiovascular events (MACE) [15]. The association of high ABPI with cardiovascular events in this high-risk population has not been previously established. This study aimed to clarify the risk factors and prognostic implications of a high ABPI in a hospital population with established vascular disease. A heterogeneous group of people with established PAD attending vascular laboratories at the included sites were studied. Unlike prior studies, participants with high ABPI were separately compared to those with normal or low ABPI in order to identify the unique prognostic significance of a high ABPI within a population with PAD.

This study had two aims, firstly, to identify risk factors associated with high ABPI, and secondly, to compare the risk of MACE, amputation and all-cause mortality for PAD patients with high ABPI, to those of PAD patients with normal or low ABPI.

## Methods

### Study design and participants

This investigation was designed as part of an ongoing prospective cohort study that commenced in 2002 [16, 17]. Participants with PAD were recruited from out-patient clinics at two public (Townsville University and Royal Brisbane and Women’s Hospitals) and two private (the Mater Hospital Townsville and Gosford Vascular Services) vascular departments between February 2002 and November 2019. Participants with the following types of PAD were eligible for inclusion: a) Asymptomatic carotid artery stenosis defined as the presence of ≥50% stenosis or occlusion of at least one carotid artery identified by carotid duplex but the absence of physician confirmed symptoms of focal transient ischemic attack, amaurosis fugax or stroke [18]; b) Intermittent claudication, with clinical or imaging evidence of lower limb athero-thrombosis, but no symptoms of rest pain or tissue loss [19]; c) Aneurysm of the aorta or peripheral arteries defined as previously reported [16, 20]; d) Symptomatic carotid artery stenosis: Defined as the presence of ≥50% stenosis or occlusion of at least one carotid artery identified with carotid duplex with the presence of physician confirmed symptoms of focal transient ischemic attack, amaurosis fugax or stroke [18]; e) Rest pain or tissue loss of the lower limb [19]. Written informed consent was obtained from all participants upon entry into the study. Patients in whom ABPI could not be measured at the time of recruitment were excluded from the study. The study was performed in accordance with the declaration of Helsinki and ethical approval was granted from Townsville Hospital and Health Services Ethics Committee.

### Definitions of risk factors and medications recorded

Age and sex were recorded at recruitment. Current smoking was defined as smoking within the last month, past smoking as having given up regularly smoking more than one month ago and never smoking as no prior history of smoking [16, 21]. Hypertension, diabetes and stroke were defined by a documented past history of diagnosis of these conditions [16, 21]. Ischemic heart disease (IHD) was defined as a documented history of MI, angina or previous treatment of IHD [21]. End-stage renal failure was defined by the requirement for renal dialysis. All prescribed medications including anti-platelet drugs, anti-coagulants, statins, fibrates, angiotensin converting enzyme (ACE) inhibitors, angiotensin receptor blockers, calcium channel blockers, furosemide, insulin, metformin and other hypoglycaemic medications were recorded at the time of recruitment.

### Measurement of ABPI

ABPI was measured by a trained sonographer or researcher within a vascular laboratory. Participants rested supine for 10 minutes and ABPI was assessed using a 5 MHz Doppler probe and sphygmomanometer cuff, with a width at least 40% of the limb circumference, according to guidelines [10, 22]. ABPI was reported in each leg as the maximum of dorsalis pedis or posterior tibial divided by the maximum brachial pressure on either side. Subsequently ABPI was classified as normal (0.91–1.39 in both legs), high (≥1.4 in either leg), low (0.5–0.9 in at least one leg but not ≥1.4 in either leg) or very low (<0.5 in at least one leg but not ≥1.4 in either leg) [10].

### Definition and assessment of outcomes

Participants were followed up as part of normal care according to local clinical policies. Participants were offered at least one follow-up appointment and usually followed up annually. Outcome data were recorded during clinical reviews on case report forms. Hospital charts and electronic records were also reviewed by a vascular specialist. Outcome data were also obtained from linked hospital admission records using the Queensland hospital admitted patient data collection as previously described [16, 17, 23, 24]. This data collection is regularly audited to minimize inaccuracies [25]. The primary outcome of this study was MACE incidence, defined as the first occurrence of MI, stroke or cardiovascular death. Secondary outcomes included the individual incidence of MI or stroke alone, any lower limb amputation, major lower limb amputation (defined as above the ankle) alone and all-cause mortality.

### Sample size

It was aimed to have adequate power to test the hypothesis that high, compared to normal or low, ABPI was associated with an increased hazard of MACE. Prior studies suggest that MACE is common in people with PAD occurring in between 30 and 40% during short term follow-up [16, 19, 23, 26]. Monte-Carlo simulations suggest that a multivariable regression model is powered sufficiently when 10 outcome events per degree of freedom of the predictor variables are observed [27]. It was estimated that the two year incidence of MACE would be approximately 30% and planned to adjust for 13 variables, some with multiple degrees of freedom, including age, sex, presentation (asymptomatic carotid stenosis, intermittent claudication, aneurysm of the aorta or peripheral arteries, symptomatic carotid stenosis or tissue loss or rest pain), current smoking, diabetes, hypertension, IHD, stroke, end-stage renal failure, prescription of statins, anti-coagulants, anti-platelets and furosemide. Based on these estimates it was felt that a sample size of about 1500 participants would be well powered to test the main hypothesis through expected a total of over 400 primary outcome events, i.e. well in excess of the 10 outcome events per variable in the Cox proportional hazard analysis. This was expected to allow appropriate power for analyses limited to sub-groups of participants as well.

### Data analysis

The characteristics of participants were compared in relation to their ABPI group (normal, low, very low or high). Continuous data were not normally distributed, as confirmed using the Shapiro Wilk test and were presented as median and inter-quartile range (IQR) and compared between groups using the Kruskal-Wallis test. Categorical variables were compared using Pearson’s chi squared test. Kaplan Meier analysis was used to calculate the observed incidence of clinical events and the log rank test to statistically compare incidence rates. Cox proportional hazard analyses assessed the association of high ABPI, compared to normal or low or very low ABPI combined (≤0.90), with events adjusted for other risk factors including age, sex, presentation (asymptomatic carotid stenosis, intermittent claudication, AAA, symptomatic carotid stenosis or tissue loss or rest pain), current smoking, diabetes, hypertension, IHD, stroke, end-stage renal failure, prescription of statins, anti-coagulants, anti-platelets and furosemide. A further analysis was performed to examine the association of high ABPI with events when comparing separately to people with low (0.50–0.90) or very low (<0.50) ABPI. A sensitivity analysis was performed to examine the effect of excluding people in whom ABPI was only measured in one leg on the main Cox proportional hazard analysis findings. All adjusted analyses were stratified by presenting complaint and end-stage renal failure in order to comply with model assumptions. Participants were censored at the time of their first relevant event, or at the date of last follow-up if no event was experienced. All presented model conformed to the proportional hazards assumption demonstrated by a global p-value >0.05. Hazard ratios and 95% confidence intervals (CI) were presented. The total number of events experienced by the patients in different ABPI groups was also quantified. Participants were not censored at the time of first event for these analyses. Data were analysed using the SPSS v 25 (IBM, Armonk, NY) and R software packages. P values of <0.05 were accepted to be significant for all analyses.

## Results

### Risk factors associated with high ABPI

Of a total of 1533 participants that had ABPI measured in both (n = 1413) or one leg (n = 120; 54 left only; 66 right only), 104 (6.8%) had a high ABPI (Table 1). Sex, presenting problem, risk factors and medications varied significantly according to ABPI group (see Table 1).

**Table 1:** Risk factors and medications in relation to ankle-brachial pressure index amongst people with peripheral artery disease.

Risk factor	Ankle-brachial pressure index				
	Normal	Low	Very low	High	P value
Participants	596	676	157	104	
Age (years)	70 (63–76)	69 (62–75)	70 (64–75)	69 (62–76)	0.668
Male	461 (77.4)	511 (75.6)	104 (66.2)	77 (74.0)	0.039
**Presenting problem**					<0.001
Asymptomatic carotid artery stenosis	38 (6.4)	25 (3.7)	3 (1.9)	3 (2.9)	
Intermittent claudication	123 (20.6)	370 (54.7)	85 (54.1)	50 (48.1)	
Aortic or peripheral aneurysm	297 (49.8)	168 (24.9)	24 (15.3)	19 (18.3)	
Symptomatic carotid artery stenosis	38 (6.4)	29 (4.3)	16 (10.2)	8 (7.7)	
Tissue loss or rest pain	100 (16.8)	84 (12.4)	29 (18.5)	24 (23.1)	
**Documented past history**					
Current smoking	150 (25.2)	241 (35.7)	66 (42.0)	13 (12.5)	<0.001
Past smoking	309 (51.8)	369 (54.6)	76 (42.0)	71 (68.3)	
Hypertension	427 (71.6)	496 (73.4)	134 (85.4)	87 (83.7)	0.001
Diabetes	216 (36.2)	209 (30.9)	50 (31.8)	58 (55.8)	<0.001
Ischemic heart disease	255 (42.8)	299 (44.2)	73 (46.5)	67 (64.4)	0.001
Stroke	48 (8.1)	67 (9.9)	21 (13.4)	19 (18.3)	0.007
End-stage renal failure	5 (0.8)	1 (0.2)	1 (0.6)	6 (5.8)	<0.001
**Medication**					
Any anti-platelet	369 (61.9)	490 (72.5)	131 (83.4)	74 (71.2)	<0.001
Any anti-coagulant	54 (9.1)	42 (6.2)	12 (7.6)	16 (15.4)	0.010
Statin	406 (68.1)	460 (68.0)	116 (73.9)	80 (76.9)	0.153
Fibrate	22 (3.7)	24 (3.6)	7 (4.5)	2 (1.9)	0.753
Angiotensin converting enzyme inhibitor	207 (34.7)	270 (39.9)	69 (43.9)	49 (47.1)	0.025
Angiotensin receptor blocker	153 (25.7)	161 (23.8)	44 (28.0)	26 (25.0)	0.703
Calcium channel blocker	152 (25.5)	210 (31.1)	61 (38.9)	35 (33.7)	0.005
Furosemide	41 (6.9)	62 (9.2)	19 (12.1)	18 (17.3)	0.003
Metformin	148 (24.8)	126 (18.6)	31 (19.7)	36 (34.6)	0.001
Insulin	51 (8.6)	45 (6.7)	13 (8.3)	22 (21.2)	<0.001
Any other hypoglycemic	79 (13.3)	89 (13.2)	16 (10.2)	31 (29.8)	<0.001

Shown are number (percentage) or median (inter-quartile range). Ankle-brachial pressure index group: 0.91–1.39 = normal; 0.50–0.90 = low; <0.50 = very low; ≥1.40 = high.

### Association of high, compared with normal, ABPI with adverse events

**Fig 1:**
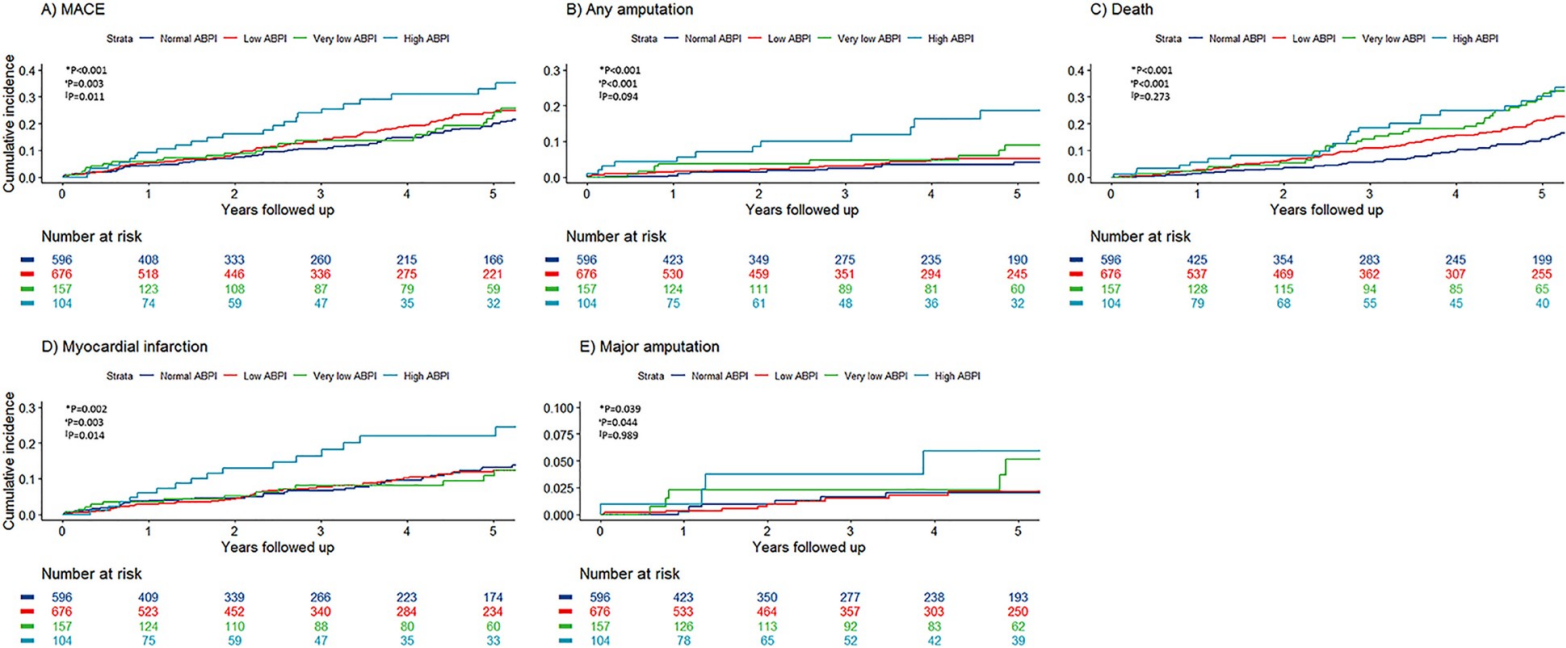
Incidence of major cardiovascular events. (a), any amputation (b), death (c), myocardial infarction (d) and major amputation (e) in participants with high (light blue line) compared to those with normal (dark blue line), low (red line) or very low (green line) ankle-brachial pressure index. * Denotes statistical comparisons between participants with high and normal ankle-brachial pressure index. + Denotes statistical comparisons between participants with high and low ankle-brachial pressure index. ^‖^ Denotes statistical comparisons between participants with high and very low ankle-brachial pressure index.

Participants were followed for a median of 3.3 (1.0–7.1) years. During this period 389 (25.4%) participants’ died and 172 (11.2%), 78 (5.1%) and 76 (5.0%) had at least one MI, stroke and amputation, respectively. 142 participants (9.3%) did not attend any follow-up appointments and were considered lost to follow-up. Participants with a high ABPI had a higher incidence of MACE, MI, any amputation and death than those with a normal ABPI both before and after adjustment for other risk factors (Table 2 and Fig 1). High, compared to normal, ABPI was associated with a higher incidence of major amputation in before but not after adjusting for other risk factors (Table 2 and Fig 1).

**Table 2:** Association of high ankle-brachial pressure index with adverse events.

Events	Compared to participants with normal ABPI		Compared to participants with low or very low ABPI	
Models	Unadjusted	Adjusted*	Unadjusted	Adjusted*
MACE	**2.15 (1.45, 3.19)**	**1.69 (1.07, 2.65)**	**1.75 (1.21, 2.52)**	**1.51 (1.02, 2.23)**
Myocardial infarction	**2.18 (1.30, 3.64)**	**1.93 (1.07, 3.46)**	**2.10 (1.29, 3.41)**	**1.92 (1.16, 3.19)**
Stroke	1.48 (0.64, 3.41)	1.19 (0.47, 3.05)	1.38 (0.62, 3.07)	1.59 (0.68, 3.71)
Any amputation	**4.45 (2.16, 9.17)**	**3.51 (1.47, 8.39)**	**2.77 (1.49, 5.14)**	1.84 (0.92, 3.69)
Major amputation	**2.99 (1.00, 8.92)**	3.24 (0.72, 14.62)	2.06 (0.78, 5.46)	1.65 (0.51, 5.28)
All-cause mortality	**2.13 (1.47, 3.08)**	**1.67 (1.09, 2.56)**	**1.56 (1.11, 2.20)**	**1.47 (1.02, 2.14)**

Shown are hazard ratios (95% confidence intervals) for events in comparison to participants with normal or low ABPI. MACE = Major adverse cardiovascular events.

* Adjusted for age, sex, presenting problem, current smoking, diabetes, hypertension, ischemic heart disease, stroke, end-stage renal failure, prescription of statins, anti-coagulants, anti-platelet and furosemide medications.

### Association of high, compared with low, ABPI with adverse events

Participants with a high ABPI had a higher risk of MACE, MI and death than those with low or very low ABPI combined (≤0.90), before and after adjustment for other risk factors (Table 2 and Fig 1). Participants with a high ABPI had a higher risk of any amputation than those with low or very low ABPI combined (≤0.90), before but not after adjustment for other risk factors (Table 2 and Fig 1). Participants with high ABPI had a higher risk of MI, any amputation and death, than those with low ABPI (0.50–0.90), both before and after adjusting for other risk factors (Table 3 and Fig 1). Participants with high ABPI had a higher risk of MACE than those with low ABPI (0.50–0.90), before but not after adjusting for other risk factors (Table 3 and Fig 1). Participants with high ABPI had a higher risk of MACE and MI, but not stroke, amputation or death, than those with very low ABPI (<0.50), both before and after adjusting for other risk factors (Table 3 and Fig 1).

**Table 3:** Association of high ankle-brachial pressure index with adverse events.

Events	Compared to participants with low ABPI		Compared to participants with very low ABPI	
Models	Unadjusted	Adjusted*	Unadjusted	Adjusted*
MACE	**1.74 (1.20, 2.52)**	1.46 (0.98, 2.19)	**1.82 (1.14, 2.92)**	**1.88 (1.11, 3.21)**
Myocardial infarction	**2.07 (1.27, 3.40)**	**1.94 (1.15, 3.29)**	**2.21 (1.16, 4.22)**	**2.43 (1.18, 4.97)**
Stroke	1.45 (0.64, 3.27)	1.55 (0.64, 3.78)	1.14 (0.44, 2.97)	1.31 (0.44, 3.89)
Any amputation	**3.09 (1.62, 5.89)**	**2.19 (1.06, 4.55)**	1.91 (0.88, 4.15)	1.20 (0.47, 3.09)
Major amputation	2.76 (0.98, 7.74)	2.60 (0.73, 9.26)	1.01 (0.34, 3.03)	0.91 (0.23, 3.65)
All-cause mortality	**1.67 (1.18, 2.37)**	**1.54 (1.04, 2.26)**	1.26 (0.83, 1.90)	1.31 (0.82, 2.11)

Shown are hazard ratios (95% confidence intervals) for events in comparison to participants with normal or low ABPI. MACE = Major adverse cardiovascular events.

* Adjusted for age, sex, presenting problem, current smoking, diabetes, hypertension, ischemic heart disease, stroke, end-stage renal failure, prescription of statins, anti-coagulants, anti-platelet and furosemide medications.

### Sensitivity analysis

In analyses excluding the 120 participants that had ABPI only measured in one leg, the only notable change from the main analysis was that the risk of MI alone was no longer significantly higher in participants with high compared to normal ABPI, after adjusting for other risk factors (Table 4).

**Table 4:** Association of high ankle-brachial pressure index with clinical events excluding participants in whom ABPI was only measured in one leg.

Events	Compared to participants with normal ABPI		Compared to participants with low or very low ABPI	
Models	Unadjusted	Adjusted*	Unadjusted	Adjusted*
MACE	**2.25 (1.50, 3.38)**	**1.68 (1.04, 2.71)**	**1.85 (1.27, 2.68)**	**1.68 (1.12, 2.51)**
Myocardial infarction	**2.22 (1.32, 3.74)**	1.78 (0.97, 3.24)	**2.16 (1.33, 3.51)**	**2.15 (1.29, 3.60)**
Stroke	1.58 (0.67, 3.70)	1.24 (0.46, 3.32)	1.52 (0.68, 3.40)	1.66 (0.70, 3.92)
Any amputation	**4.20 (1.84, 9.58)**	**3.50 (1.26, 9.69)**	**2.35 (1.17, 4.71)**	1.89 (0.87, 4.11)
Major amputation	2.49 (0.62, 9.96)	1.81 (0.27, 11.90)	1.35 (0.40, 4.55)	1.44 (0.38, 5.53)
All-cause mortality	**2.20 (1.48, 3.28)**	**1.72 (1.08, 2.75)**	**1.52 (1.06, 2.19)**	**1.52 (1.02, 2.25)**

Shown are hazard ratios (95% confidence intervals) for events in comparison to participants with normal or low ABPI. MACE = Major adverse cardiovascular events.

* Adjusted for age, sex, presenting problem, current smoking, diabetes, hypertension, ischemic heart disease, stroke, end-stage renal failure, prescription of statins, anti-coagulants, anti-platelet and furosemide medications.

### Association of high ABPI with total clinical events

Many participants had multiple clinical events. Table 5 illustrates the total number of clinical events and the number of participants that had the events in relation to ABPI group. There was a marked excess of total MACE, MI and amputation in participants with high ABPI. There were, for example, a total of 43 MIs in 104 participants with high ABPI compared with 123 in 676 participants with low ABPI (Table 5).

**Table 5:** Total clinical events in relation to ankle-brachial pressure index.

	Normal	Low	Very low	High
Participants	596	676	157	104
Follow-up (years)	2.7 (0.6–6.4)	3.4 (1.3–7.6)	4.4 (1.9–7.5)	3.2 (1.0–7.0)
Total number of MACE	141	257	70	69
Number of participants that the MACE events occurred in	95 (15.9)	161 (23.8)	40 (25.5)	34 (32.7)
Total myocardial infarction events	71	123	29	43
Number of participants that the myocardial infarction events occurred in	55 (9.2)	78 (11.5)	19 (12.1)	20 (19.2)
Total stroke events	30	39	21	8
Number of participants that the stroke events occurred in	25 (4.2)	34 (5.0)	12 (7.6)	7 (6.7)
Total amputations	33	52	21	34
Number of participants that the amputations occurred in	17 (2.9)	33 (4.9)	13 (8.3)	13 (12.5)
Total major amputations	16	14	14	8
Number of participants that the major amputations occurred in	9 (1.5)	13 (1.9)	9 (5.7)	5 (4.8)

Shown are total number of events and number (percentage) of participants that had the events from each ABPI group.

MACE = Major adverse cardiovascular events.

## Discussion

It commonly believed that an important weakness of the ABPI test is its non-diagnostic value in people with incompressible tibial arteries [10]. This study demonstrates that the finding of a high ABPI, amongst people with PAD, provides important prognostic information. High ABPI was independently associated with an increased hazard of MACE, MI and death. Strikingly, the increased hazard of these important clinical events was found in comparison to participants with low ABPI, as well as compared to those with normal ABPI. This demonstrates the clear clinical value of identifying a high ABPI in people with PAD.

In the current study, about 7% of participants had an ABPI ≥1.4, a rate higher than reported in community populations [3, 7–9]. Participants with high ABPI had different risk factors to those with normal or low ABPI, including high prevalence of diabetes, in keeping with its accepted role in promoting arterial calcification [28]. They also had a relatively low frequency of current smoking by comparison to participants with low ABPI.

Prior studies of community populations have shown independent associations of high ABPI with cardiovascular events in some but not all populations [3, 5, 7–10, 12–14]. In the current study participants were recruited from hospital out-patient clinics and had PAD diagnosed by a vascular specialist. This population included a large number of people with low ABPI which allowed comparison with that high risk group, unlike prior studies which have simply compared to people with normal ABPI [3, 5, 7–10, 12–14]. Within this PAD population, high ABPI strongly and independently predicted the key clinical events of MACE, MI and death. After adjusting for other risk factors, participants with high ABPI had an approximate 2-fold increased risk of MI compared with those with low or very low ABPI combined. This finding was similar when comparing participants with high ABPI to those with very low ABPI alone.

Despite the high incidence of events in participants with high ABPI, about 25% were not prescribed a statin and 29% not receiving any anti-platelet medication. Given the very high rate of cardiovascular events in people with high ABPI, it is likely they would substantially benefit from intensive medical management. High ABPI might identify people, for example, in whom it is highly beneficial and cost-effective to intensively lower low density lipoprotein- cholesterol with recently introduced Proprotein Convertase Subtilisin/kexin type 9 (PCSK9) Inhibitions [29].

The results of this study should be interpreted acknowledging its strengths and weakness. The study includes a heterogeneous population with multiple different presentations recruited from hospital out-patient clinics and thus is likely to be generalizable to similar heterogeneous populations that have been referred to vascular surgery out-patient clinics. The findings may not be relatable to populations recruited from the community or to homogenous presentations of PAD. While high ABPI was independently associated with cardiovascular events, it is possible that risk factors that were not measured, such as renal function, thrombophilia or previous peripheral revascularization, and therefore were unable to be adjusted for may have contributed to this association. High ABPI is a measure of arterial calcification, a recognized predictor of clinical events, and adjustment for this may have influenced the associations demonstrated [11]. ABPI is, however, more straightforward and cheaper to measure than arterial calcification. In addition, clinical outcome data were extracted from patient medical records, rather than an independent adjudication committee and the potential that some clinical events may have been missed, or misclassified cannot be excluded. Also whilst analyses were adjusted to account for potentially important differences in medication use between the groups at the time of enrolment, changes in medications during follow-up were not recorded and could not be accounted for. Finally a number of participants were lost to follow-up which may have affected findings.

In conclusion, this study demonstrates that amongst people with diagnosed PAD, a high ABPI is an important indicator of high risk of cardiovascular events, including MACE, MI and death. Importantly the risk of events was higher than for people with low ABPI. These findings suggest that people with high ABPI should be considered for intensive medical treatment to lower their risk of clinically important events.

## References

[1] Aboyans V, Ricco JB, Bartelink MEL, Bjorck M, Brodmann M, Cohnert T, et al Editor's Choice—2017 ESC Guidelines on the Diagnosis and Treatment of Peripheral Arterial Diseases, in collaboration with the European Society for Vascular Surgery (ESVS). Eur J Vasc Endovasc Surg. 2018;55(3):305–68. 10.1016/j.ejvs.2017.07.018 28851596

[2] Gerhard-Herman M, Gornik H, Barrett C, Barshes N, Corriere M, Drachman D, et al 2016 AHA/ACC Guideline on the Management of Patients With Lower Extremity Peripheral Artery Disease: A Report of the American College of Cardiology/American Heart Association Task Force on Clinical Practice Guidelines. Circulation. 2017;135(12):e726–e79. 10.1161/CIR.0000000000000471 27840333 PMC5477786

[3] Ankle Brachial Index Collaboration, Fowkes FG, Murray GD, Butcher I, Heald CL, Lee RJ, et al Ankle brachial index combined with Framingham Risk Score to predict cardiovascular events and mortality: a meta-analysis. JAMA. 2008;300(2):197–208. 10.1001/jama.300.2.197 18612117 PMC2932628

[4] Newman A, Shemanski L, Manolio T, Cushman M, Mittelmark M, Polak J, et al Ankle-arm index as a predictor of cardiovascular disease and mortality in the Cardiovascular Health Study. The Cardiovascular Health Study Group. Arterioscler Thromb Vasc Biol 1999;19(3):538–45. 10.1161/01.atv.19.3.538 10073955

[5] Hong J, Leonards C, Endres M, Siegerink B, Liman T. Ankle-Brachial Index and Recurrent Stroke Risk: Meta-Analysis. Stroke. 2016;47(2):317–22. 10.1161/STROKEAHA.115.011321 26658450

[6] Fowkes FGR, Murray GD, Butcher I, Folsom AR, Hirsch AT, Couper DJ, et al Development and validation of an ankle brachial index risk model for the prediction of cardiovascular events. Eur J Prev Cardiol 2014;21(3):310–20. 10.1177/2047487313516564 24367001 PMC4685459

[7] Allison M, Hiatt W, Hirsch A, Coll J, Criqui M. A high ankle-brachial index is associated with increased cardiovascular disease morbidity and lower quality of life. J Am Coll Cardiol 2008;51(13):1292–8. 10.1016/j.jacc.2007.11.064 18371562

[8] Criqui M, McClelland R, McDermott M, Allison M, Blumenthal R, Aboyans V, et al The ankle-brachial index and incident cardiovascular events in the MESA (Multi-Ethnic Study of Atherosclerosis). J Am Coll Cardiol 2010;56(18):1506–12. 10.1016/j.jacc.2010.04.060 20951328 PMC2962558

[9] Wattanakit K, Folsom A, Duprez D, Weatherley B, Hirsch A. Clinical significance of a high ankle-brachial index: insights from the Atherosclerosis Risk in Communities (ARIC) Study. Atherosclerosis. 2007;190(2):459–64. 10.1016/j.atherosclerosis.2006.02.039 16574125

[10] Aboyans V, Criqui MH, Abraham P, Allison MA, Creager MA, Diehm C, et al Measurement and interpretation of the ankle-brachial index: a scientific statement from the American Heart Association. Circulation. 2012;126(24):2890–909. 10.1161/CIR.0b013e318276fbcb 23159553

[11] Parr A, Buttner P, Shahzad A, Golledge J. Relation of infra-renal abdominal aortic calcific deposits and cardiovascular events in patients with peripheral artery disease. Am J Cardiol 2010;105(6):895–9. 10.1016/j.amjcard.2009.10.067 20211340

[12] Resnick H, Lindsay R, McDermott M, Devereux R, Jones K, Fabsitz R, et al Relationship of high and low ankle brachial index to all-cause and cardiovascular disease mortality: the Strong Heart Study. Circulation. 2004;109(6):733–9. 10.1161/01.CIR.0000112642.63927.54 14970108

[13] Yang Y, Liu L, Sun H, Nie F, Hu X. High ankle brachial index and cardiovascular outcomes in the general population and suspected or established cardiovascular disease patients: a meta-analysis. Int Angiol 2019: 10.23736/S0392-9590.19.04276-7 31814377

[14] Hendriks EJ, Westerink J, de Jong PA, de Borst GJ, Nathoe HM, Mali WP, et al Association of High Ankle Brachial Index With Incident Cardiovascular Disease and Mortality in a High-Risk Population. Arterioscler Thromb Vasc Biol. 2016;36(2):412–7. 10.1161/ATVBAHA.115.306657 26715681

[15] Manapurathe DT, Moxon JV, Krishna SM, Rowbotham S, Quigley F, Jenkins J, et al Cohort Study Examining the Association Between Blood Pressure and Cardiovascular Events in Patients With Peripheral Artery Disease. J Am Heart Assoc. 2019;8(6):e010748 10.1161/JAHA.118.010748 30845872 PMC6475052

[16] Golledge J, Cronin O, Iyer V, Bradshaw B, Moxon JV, Cunningham MA. Body mass index is inversely associated with mortality in patients with peripheral vascular disease. Atherosclerosis. 2013;229(2):549–55. 10.1016/j.atherosclerosis.2013.04.030 23742964

[17] Morris D, Rodriguez A, Moxon J, Cunningham M, McDermott M, Myers J, et al Association of lower extremity performance with cardiovascular and all-cause mortality in patients with peripheral artery disease: a systematic review and meta-analysis. J Am Heart Assoc 2014;3(4):e001105 10.1161/JAHA.114.001105 25122666 PMC4310407

[18] Palamuthusingam D, Quigley F, Golledge J. Implications of the finding of no significant carotid stenosis based on data from a regional Australian vascular unit. Ann Vasc Surg. 2011;25(8):1050–6. 10.1016/j.avsg.2011.05.015 21831585

[19] Golledge J, Moxon JV, Rowbotham S, Pinchbeck J, Yip L, Velu R, et al Risk of major amputation in patients with intermittent claudication undergoing early revascularization. Br J Surg. 2018;105(6):699–708. 10.1002/bjs.10765 29566427

[20] Magee R, Quigley F, McCann M, Buttner P, Golledge J. Growth and risk factors for expansion of dilated popliteal arteries. Eur J Vasc Endovasc Surg 2010;39(5):606–11. 10.1016/j.ejvs.2009.12.031 20122854

[21] Golledge J, Leicht A, Crowther RG, Clancy P, Spinks WL, Quigley F. Association of obesity and metabolic syndrome with the severity and outcome of intermittent claudication. Journal of Vascular Surgery. 2007;45(1):40–6. 10.1016/j.jvs.2006.09.006 17123770

[22] Fernando M, Crowther R, Cunningham M, Lazzarini P, Sangla K, Golledge J. Lower limb biomechanical characteristics of patients with neuropathic diabetic foot ulcers: the diabetes foot ulcer study protocol. BMC Endocr Disord 2015;15:59 10.1186/s12902-015-0057-7 26499881 PMC4619003

[23] Morris DR, Singh TP, Moxon JV, Smith A, Stewart F, Jones RE, et al Assessment and validation of a novel angiographic scoring system for peripheral artery disease. British Journal of Surgery. 2017;104(5):544–54. 10.1002/bjs.10460 28140457

[24] Moxon JV, Jones RE, Wong G, Weir JM, Mellett NA, Kingwell BA, et al Baseline serum phosphatidylcholine plasmalogen concentrations are inversely associated with incident myocardial infarction in patients with mixed peripheral artery disease presentations. Atherosclerosis. 2017;263:301–8. 10.1016/j.atherosclerosis.2017.06.925 28728066

[25] Queensland Health. Queensland hospital admitted data collection manual 2015–2016 [Available from: https://www.health.qld.gov.au/hsu/collections/qhapdc.

[26] Morris DR, Skalina TA, Singh TP, Moxon JV, Golledge J. Association of Computed Tomographic Leg Muscle Characteristics With Lower Limb and Cardiovascular Events in Patients With Peripheral Artery Disease. J Am Heart Assoc. 2018;7(20):e009943 10.1161/JAHA.118.009943 30371256 PMC6474956

[27] Peduzzi P, Concato J, Kemper E, Holford TR, Feinstein AR. A simulation study of the number of events per variable in logistic regression analysis. J Clin Epidemiol. 1996;49(12):1373–9. 8970487 10.1016/s0895-4356(96)00236-3

[28] Lanzer P, Boehm M, Sorribas V, Thiriet M, Janzen J, Zeller T, et al Medial vascular calcification revisited: review and perspectives. Eur Heart J 2014;35(23):1515–25. 24740885 10.1093/eurheartj/ehu163 PMC4072893

[29] Bonaca MP, Nault P, Giugliano RP, Keech AC, Pineda AL, Kanevsky E, et al Low-Density Lipoprotein Cholesterol Lowering With Evolocumab and Outcomes in Patients With Peripheral Artery Disease: Insights From the FOURIER Trial (Further Cardiovascular Outcomes Research With PCSK9 Inhibition in Subjects With Elevated Risk). Circulation 2018;2018(137):4. 10.1161/CIRCULATIONAHA.117.032235 29133605

